# Law and Medicine

**Published:** 1874-04

**Authors:** 


### Selections.

### LAW AND MEDICINE.

Smart young limbs of the law take great delight in puzzling a medical witness when he gets on stand. One such was cross-examining the celebrated French chemist Orfila, and put to him the question whether he could state the precise amount of arsenic requisite to kill a fly. "Certainly," replied the expert; "but I must know beforehand the age of the fly, its sex, its temperament, its condition, and habits of body, whether married or single, widow or maiden, widower or bachelor."

